# Winter Retreat for Consumptives

**Published:** 1867-12

**Authors:** 


WINTER RETREAT FOR CONSUMPTIVES.

A large house is being fitted up under the superintendence of Dr. Prince, in Jacksonville, heated by furnaces, so arranged as to admit of the purification and medication of the air of the house, producing an artificial climate, not only favorable for the most speedy recovery of patients undergoing surgical treatment, but also adapted to secure the best atmosphere for those affected or threatened with pulmonary complaints.

It is expected that the repairs and alterations necessary for this purpose will be completed by the first of December.

Address DAVID PRINCE, M.D., dec1t. *Jacksonville, Ill.*

